# Esophageal Varices Presenting With Massive Hematemesis in a Chronic Alcoholic: A Case Report on a Rare Condition

**DOI:** 10.7759/cureus.63889

**Published:** 2024-07-05

**Authors:** Suhas Tivaskar, Rajasbala Dhande, Gaurav V Mishra, Anurag Luharia, Shreya Naik, Albert P Varghese, Syed Asrar Ul Haq Andrabi

**Affiliations:** 1 Radiology, Datta Meghe Institute of Higher Education & Research, Wardha, IND; 2 Radiodiagnosis, Datta Meghe Institute of Higher Education & Research, Wardha, IND; 3 Radiology, School of Allied Health Sciences, Datta Meghe Institute of Higher Education & Research, Wardha, IND

**Keywords:** endoscopic sclerotherapy, hepatosplenomegaly, hvpg, oesophageal varices, cirrhosis

## Abstract

Esophageal varices are life-threatening complications in which the enlargement of the esophageal veins causes bleeding and reduces blood flow to the esophagus. They are complications caused by portal hypertension, renal failure, hepatic dysfunction, and infection. The leading cause of esophageal varices is cirrhosis, as patients with this disease are more susceptible to forming esophageal varices. Bleeding episodes occur due to the rupture of the blood vessels. We present the case of a 45-year-old male patient in the hospital with a history of chronic alcohol use and clinical symptoms of hematemesis, a distended abdomen, and melena. The patient experienced mild symptoms of giddiness and dizziness after undergoing various radiological investigations, laboratory tests, ultrasonography (USG), and CT scans. USG diagnosed portal hypertension, gross ascites, pleural effusion, and hepatosplenomegaly. A CT scan diagnosed the patient with esophageal varices and testicular carcinoma. Laboratory tests diagnosed anemia. The treatment plan included oral and intravenous iron supplements, blood transfusions, vitamin B12, folate supplements, and nonselective beta-blockers to manage portal hypertension and reduce variceal bleeding risk. During acute bleeding episodes, vasoconstrictors and endoscopic band ligation were employed. Regular endoscopies and hepatic venous catheterization were conducted to monitor and manage the condition. Follow-up included regular assessments of hemoglobin levels, iron status, liver function tests, and periodic endoscopies. The patient’s adherence to beta-blockers was closely monitored. Esophageal varices, often resulting from portal hypertension because of cirrhosis, require early diagnosis and a combination of pharmacological and endoscopic treatments to prevent complications. Advances in treatment have reduced mortality rates, but effective management of portal hypertension and liver dysfunction remains crucial.

## Introduction

Esophageal varices are a complication caused by portal hypertension. Enlargement of the veins in the esophageal region is reported in the condition of varices. Varices generally occur in the esophagus, duodenum, and paraumbilical region. The leading causes of esophageal varices are cirrhosis and portal hypertension, because patients with these diseases are more susceptible to forming esophageal varices. Portal hypertension involves the increased influx of blood in the portal vein and the outrush. Hepatic venous catheterization is used to calculate portal hypertension. The range of portal hypertension, including its diameter and variceal size, determines the severity of the esophageal varices. This increased portal pressure leads to the condition of varices. If the hepatic venous pressure gradient (HVPG) exceeds 12 mm Hg, it causes varices [1].

Varices vary according to their size. Enlargement of esophageal varices increases the risk of bleeding and rupture of the veins. If these varices lead to bleeding and rupturing, it can cause the patient's death. The rupture of the veins causes bleeding and leads to a condition known as hematemesis. The severity should be determined according to the size of the esophageal varices. Patients with cirrhosis are most susceptible to developing esophageal varices because cirrhosis is a severe liver condition often caused by excessive alcohol consumption. Patients with cirrhosis should undergo investigative procedures for esophageal varices. The screening method for esophageal varices is endoscopy. Along with endoscopy, ultrasound findings and biochemical parameters are beneficial for diagnosing varices. The outcomes of endoscopies and other laboratory tests help diagnose esophageal varices. In biochemical parameters, platelet count should be considered when diagnosing varices because the condition affects platelet count. The diameters of the spleen and liver are also affected by esophageal varices and should be considered in the diagnosis [2].

In cirrhosis, the liver deteriorates, resulting in the formation of fibrous tissue, disarrangement of the liver’s echotexture, and portal hypertension. Portal hypertension leads to fluid formation and complications known as ascites, hepatic encephalopathy, and esophageal varices. This occurs in patients with a history of chronic alcohol consumption, which worsens liver conditions and leads to hypertension and esophageal varices. Patients with large esophageal varices experience episodes of bleeding. Increased bleeding episodes raise the risk of mortality. This bleeding results in hematemesis and causes anemia in patients [3].

## Case presentation

Patient information

We present the case of a 45-year-old male patient who came to the medicine department of our hospital. The patient arrived with a chief complaint of esophageal varices. Initially, he presented with the primary symptoms of dizziness and vomiting, which had been troubling him significantly. Clinically, the patient exhibited signs of hematemesis, having experienced two episodes within the past week, along with melena. He reported that these distressing symptoms had begun within the last month, indicating the recent onset of his current health issues.

Medical and surgical history

The patient’s medical history revealed a long-standing history of chronic alcohol consumption spanning many years. Specifically, he consistently consumed 120 ml of alcohol daily, according to the detailed account of his history. In addition to his alcohol consumption, the patient was diagnosed with anemia a year ago, a condition that has likely impacted his overall health. Notably, there was no history of diabetes, heart disease, or asthma. Furthermore, the patient had no history of any gastrointestinal surgeries or other significant surgical interventions, indicating that he had not undergone any major procedures that could have contributed to his current health status.

Physical examination and investigation

Upon admission, a thorough physical examination was conducted. The patient was febrile, presenting with a slightly increased pulse rate, an average respiratory rate, and higher-than-usual blood pressure. His BMI was calculated at 26.99, with a height of 170 cm and a weight of 78 kg. Importantly, there were no signs of pallor, icterus, clubbing, cyanosis, or edema. His family history did not reveal any significant health issues. The patient’s blood group was identified as O Rh positive. Laboratory tests indicated thrombocytopenia, and he was diagnosed with anemia. Further investigations confirmed the presence of esophageal varices. Consequently, the doctor recommended several diagnostic procedures.

**Figure 1:**
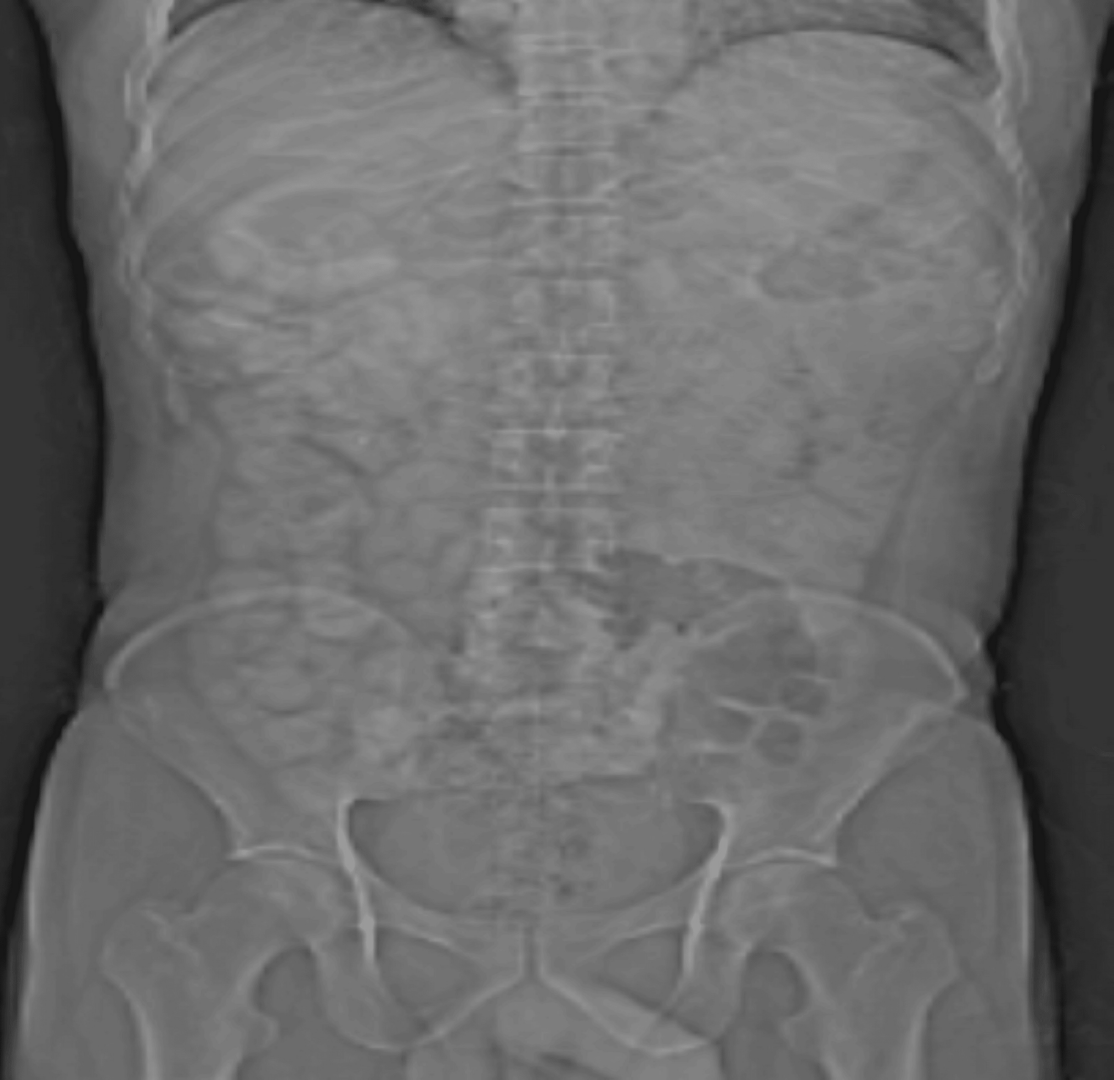
Abdomen radiograph showing bowel distention with mild ascites

The doctor recommended obtaining chest and abdomen X-rays for radiological evaluation. In a previous investigation, the chest radiograph revealed pleural effusion, while the abdominal radiograph showed signs of abdominal distention, commonly known as ascites, as illustrated in Figure 1.

To further confirm the medical condition, the doctor suggested a barium swallow procedure performed in the Trendelenburg position, also known as the low head position. This procedure revealed half-round and full-round impressions in the lower one-third of the esophagus. Additionally, a previous investigation revealed hepatosplenomegaly, including USG of the abdomen and pelvis.

**Figure 2:**
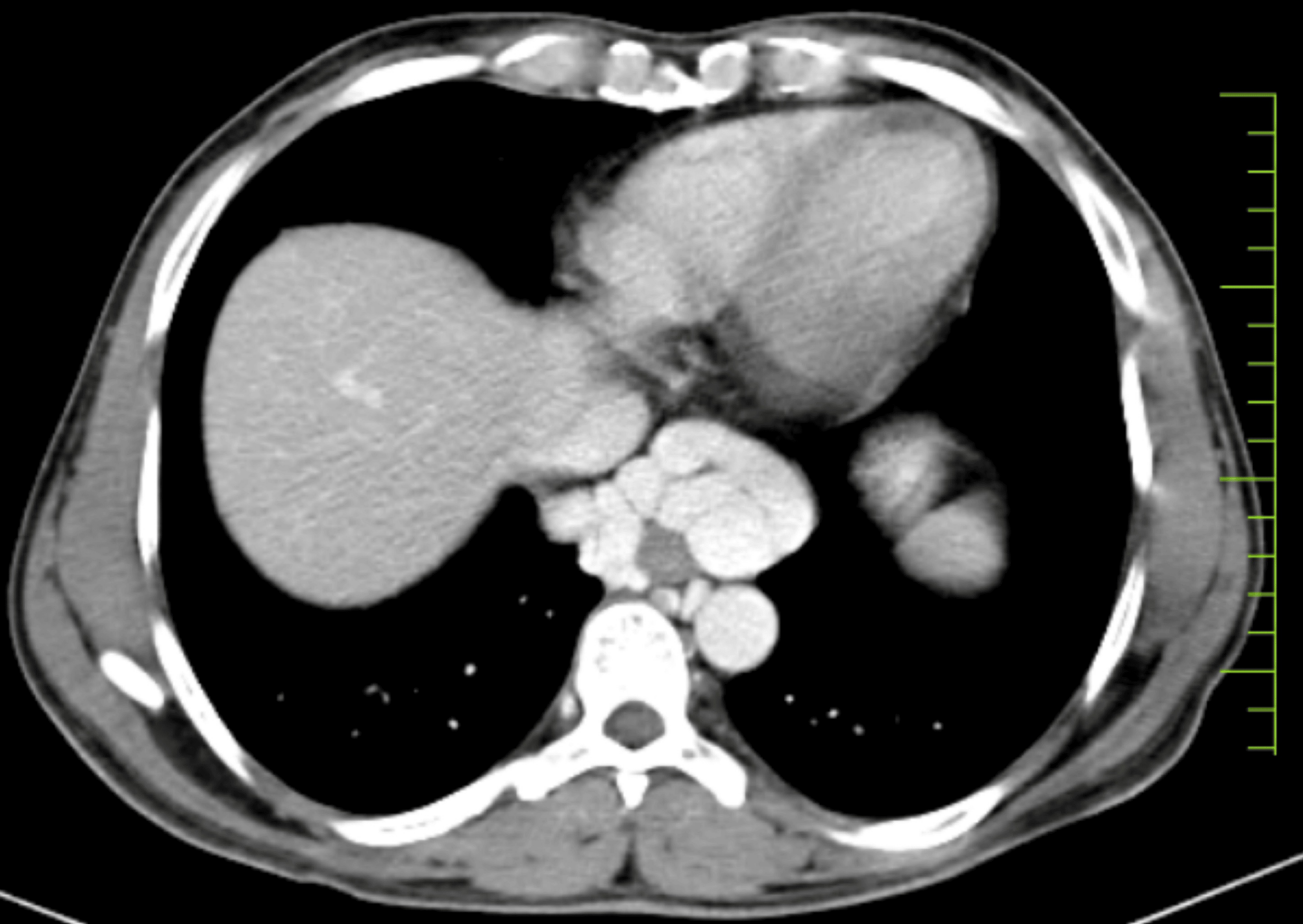
Axial CT scan image showing the expansion of esophageal varices toward the stomach

Further findings included dilated and tortuous splenic and portal veins, with ascites also confirmed in the X-ray report. The doctor recommended a CT scan of the thorax and abdomen with contrast for a more detailed evaluation. The contrast-enhanced CT scan of the thorax and abdomen/pelvis confirmed pleural effusion and hepatosplenomegaly. Additionally, the reports noted dilation and tortuosity of the splenic vein, portal vein dilation, ascites, and prominent esophageal varices, as illustrated in Figure 2.

Treatment

The patient adhered to a comprehensive treatment plan for managing esophageal varices and anemia. A regimen of oral iron supplements was prescribed to address the anemia, including Fer XT, Ferrous Ascorbate 100 mg, Folic Acid 500 mcg, and zinc sulfate tablets. In cases where oral supplements were ineffective or not well tolerated, intravenous iron therapy was considered. Additionally, blood transfusions were administered to elevate hemoglobin levels and stabilize the patient during acute episodes. Vitamin B12 and folate supplements were also given to rectify underlying nutritional deficiencies. Throughout the treatment, the patient’s hemoglobin levels and iron status were closely monitored to assess the treatment’s effectiveness and make any necessary adjustments.

For the management of esophageal varices, nonselective beta-blockers such as propranolol or nadolol were prescribed to decrease portal hypertension and reduce the risk of variceal bleeding. During acute bleeding episodes, vasoconstrictors like octreotide were used to control hemorrhage. Additionally, the patient underwent endoscopic band ligation (EBL), a procedure in which elastic bands are placed around the varices to prevent bleeding. Regular endoscopies were performed to evaluate the condition of the esophageal varices. To accurately measure portal hypertension, hepatic venous catheterization was conducted, revealing an HVPG exceeding 12 mm Hg. The presence of esophageal varices was confirmed by these diagnostic procedures, and the treatment plan was adjusted accordingly to manage the condition effectively.

Follow-up

The patient had regular follow-up appointments to monitor treatment progress for anemia and esophageal varices. Periodic checks of hemoglobin levels, iron studies, and liver function tests were conducted to evaluate the effectiveness of iron supplementation and adjust the treatment as needed. Additionally, endoscopic examinations were scheduled every six to 12 months to monitor the varices’ condition and perform further band ligation or sclerotherapy if necessary.

The patient’s adherence to nonselective beta-blockers was closely monitored, with dosage adjustments made based on blood pressure and heart rate to ensure optimal management of portal hypertension. These regular assessments and adjustments were crucial in providing comprehensive care, ensuring the patient’s condition was managed effectively, and preventing complications associated with anemia and esophageal varices.

## Discussion

Esophageal varices are complications influenced by various causative factors and diagnostic methods. Portal hypertension involves the increased influx and outflow of blood in the portal vein. Portal pressure should be calculated using hepatic venous catheterization. The severity of portal hypertension determines the seriousness of esophageal varices. This increased portal pressure leads to the formation of varices. If the HVPG exceeds 12 mm Hg, it causes varices [1]. In cirrhosis patients, there are several ways to diagnose esophageal varices. The main methods are invasive and noninvasive. Platelet count and spleen ratio are noninvasive tests for diagnosing esophageal varices. There are two types of cirrhosis: compensated and decompensated. The risk of spreading and developing esophageal varices is higher in decompensated cirrhosis [2]. In the pathophysiology of esophageal varices, vasoactive mediators are released during the septic process, altering blood vessel tone. These mediators can act as vasodilators or vasoconstrictors. In esophageal varices, nitric oxide is a potent vasodilator, regulating intrahepatic vascular tone. Nitric oxide is responsible for increasing portal pressure in permeate rat liver, and enhancement occurs after nitric oxide inhibition, where it acts as a regulating vasoactive mediator in regular processes [4]. In cirrhotic liver patients, there is a lack of nitric oxide. Therefore, it cannot compensate for the vasoconstriction system [5]. Nitric oxide plays a crucial role in modulating the hemodynamics of portal hypertension. It induces vasodilation, whereas, in the splanchnic region, there is a higher occurrence of vasoconstriction in portal hypertension conditions [6].

The evolution of portal-systemic collateral occurs in portal hypertension. Consequently, gastric varices and esophageal varices can occur, with the severity of the variceal rupture determining the complexity of the condition. This complication is the most deadly and complicated in cirrhosis. Nearly 100% of cirrhotic patients are affected, with 90% having a chance of developing esophageal varices, among whom 40% are at risk of rupture and bleeding [7]. After diagnosing esophageal varices, 15-30% of patients not expected to have ruptures may be at risk of damage. The risk of rupture depends on the size of the varices and their impact on the hepatic system. Even if the patient’s hepatic condition is severe, small varices can still cause bleeding [8]. Esophageal varices are common in many alcoholic patients, with 90% also having cirrhosis. While it can be cured, once the variceal size increases and rupture bleeding occurs, it becomes the most probable cause of mortality in alcoholic patients [9]. The mortality rate has decreased over decades due to advancements in treatment.

Various effective treatments have emerged, reducing mortality rates. Endoscopic procedures, pharmacological therapies, and transjugular intrahepatic portosystemic shunt (TIPS) are among the treatments that reduce mortality and enhance medical care. By utilizing these advancements, bleeding conditions in varices can be prevented [10]. Treatment depends on the severity of the condition. Unfortunately, many patients today are succumbing to early bleeding caused by variceal rupture. Kidney failure can lead to various infections, while hepatic encephalopathy becomes a cause of mortality in esophageal varices cases. Severe portal hypertension and liver dysfunction are also factors contributing to death. Treating the liver and managing portal hypertension is essential to managing varices [11]. The primary goal of endoscopic therapies for varices is to decrease variceal wall tension by eradicating the varix. Two main methods for esophageal varices are EBL and endoscopic sclerotherapy (EST). These therapies are categorized as local treatments. They do not affect any mechanisms of pathophysiology that could lead to hypertension and variceal rupture [12]. Patients treated with either EBL or EST methods often experience a spontaneous decrease in the HVPG value, which helps prevent variceal rupture [13]. If this occurs in any patient, curing the patient with endoscopic therapies becomes easier until the varix is eradicated [14]. Patients who respond positively to endoscopic therapies have a lower probability of rebleeding, and the effectiveness of the treatment can be enhanced by adding beta-blockers alongside endoscopic therapies [15].

## Conclusions

Hepatic failure and the disease known as cirrhosis are widespread in India, with alcohol consumption being the proven cause of this deadliest disease. Cirrhosis gives rise to numerous complications, including portal hypertension, which leads to the development of a condition known as esophageal varices. Early diagnosis of bleeding and rupture should be made possible with the help of laboratory tests. Radiological imaging equipment such as X-rays, USG, and CT scans plays a crucial role in diagnosing esophageal varices and assessing their clinical condition. The HVPG should be noted to check the severity of varices. Treatment options include various pharmacological therapies, TIPSs, endoscopic therapies, and beta blockers, which can increase treatment efficiency. For prevention, every cirrhotic patient should undergo diagnostic procedures for esophageal varices.
